# Development and Use of a Real-Time Quantitative PCR Method for Detecting and Quantifying Equol-Producing Bacteria in Human Faecal Samples and Slurry Cultures

**DOI:** 10.3389/fmicb.2017.01155

**Published:** 2017-06-30

**Authors:** Lucía Vázquez, Lucía Guadamuro, Froilán Giganto, Baltasar Mayo, Ana B. Flórez

**Affiliations:** ^1^Departamento de Microbiología y Bioquímica, Instituto de Productos Lácteos de Asturias, Consejo Superior de Investigaciones Científicas, IPLA-CSICVillaviciosa, Spain; ^2^Servicio Digestivo, Hospital Universitario Central de AsturiasOviedo, Spain

**Keywords:** real time quantitative PCR, qPCR, soy isoflavones, equol, intestinal microbiology, faecal microbiota

## Abstract

This work introduces a novel real-time quantitative PCR (qPCR) protocol for detecting and quantifying equol-producing bacteria. To this end, two sets of primers targeting the dihydrodaidzein reductase (*ddr*) and tetrahydrodaidzein reductase (*tdr*) genes, which are involved in the synthesis of equol, were designed. The primers showed high specificity and sensitivity when used to examine DNA from control bacteria, such as *Slackia isoflavoniconvertens, Slackia equolifaciens, Asaccharobacter celatus, Adlercreutzia equolifaciens*, and *Enterorhabdus mucosicola*. To demonstrate the validity and reliability of the protocol, it was used to detect and quantify equol-producing bacteria in human faecal samples and their derived slurry cultures. These samples were provided by 18 menopausal women under treatment of menopause symptoms with a soy isoflavone concentrate, among whom three were known to be equol-producers given the prior detection of the molecule in their urine. The *tdr* gene was detected in the faeces of all these equol-producing women at about 4–5 log_10_ copies per gram of faeces. In contrast, the *ddr* gene was only amplified in the faecal samples of two of these three women, suggesting the presence in the non-amplified sample of reductase genes unrelated to those known to be involved in equol formation and used for primer design in this study. When *tdr* and *ddr* were present in the same sample, similar copy numbers of the two genes were recorded. However, no significant increase in the copy number of equol-related genes along isoflavone treatment was observed. Surprisingly, positive amplification for both *tdr* and *ddr* genes was obtained in faecal samples and derived slurry cultures from two non-equol producing women, suggesting the genes could be non-functional or the daidzein metabolized to other compounds in samples from these two women. This novel qPCR tool provides a technique for monitoring gut microbes that produce equol in humans. Monitoring equol-producing bacteria in the human gut could provide a means of evaluating strategies aimed at increasing the endogenous formation of this bioactive compound.

## Introduction

Epidemiological evidence suggests high intakes of soy foods or purified soy isoflavones to be associated with less intense menopause symptoms and a reduced risk of developing cardiovascular diseases, neurodegenerative diseases, and cancer (He and Chen, 2013; Wada et al., 2013; Bilal et al., 2014). Isoflavones-mediated effects appear to be driven by their hormonal (Yuan et al., 2007), antioxidant (Arora et al., 1998), and enzyme-inhibitory (Crozier et al., 2009) activities.

In soy, isoflavones are mostly found as glycoside conjugates (daidzin, genistin, and glycitin) (Franke et al., 2014). Isoflavones are more bioavailable after their deglycosylatation by cellular enzymes or enzymes belonging to certain gut bacteria (Franke et al., 2014). The aglycone moieties (daidzein, genistein, and glycitein) of isoflavone glycosides are released via the action of cellular and bacterial β-glucosidases (Islam et al., 2014). Aglycones are further metabolized into either compounds of greater biological activity or inactive molecules (Clavel and Mapesa, 2013). Equol, produced from daidzein, has the strongest oestrogenic and antioxidant activity of all isoflavone metabolites (Setchell and Clerici, 2010; Franke et al., 2014). All the animal species tested to date (including cows, pigs, sheep, chickens, mice, and rats) produce equol in response to soy (and thus daidzein) intake (Setchell and Clerici, 2010). However, only 30–60% of humans do so, and it may be only these who fully benefit from soy and/or isoflavone consumption (Franke et al., 2014).

The human gut microbiota harbours a complex and dynamic population of microorganisms, which is dominated by nutritionally-fastidious, strict anaerobic bacteria that are extremely sensitive to ambient oxygen (Thursby and Juge, 2017). Indeed, growth in culture of many of the bacterial species forming the gut microbiota has, until recently, been considered impossible (Browne et al., 2016). Therefore, culture-independent techniques are considered more suitable than the traditional methods for identifying and quantifying the components of the intestinal microbial populations (Delgado et al., 2013; Kim et al., 2015). Among the different, culture-independent, molecular techniques available, real-time quantitative PCR (qPCR) has become highly regarded as a specific detector and quantifier of microorganisms in complex microbial samples, including samples from the human gut (Furet et al., 2009; Liszt et al., 2009; Tuomisto et al., 2013; Ruengsomwong et al., 2014).

Equol biosynthesis from daidzein seems to take place through the consecutive action of three conserved reductases via dihydrodaidzein and tetrahydrodaidzein intermediates (Shimada et al., 2010, 2011; Schröder et al., 2013). Though far from complete, our knowledge of the microorganisms that produce equol from daidzein, and of the biochemical pathways involved, is growing (Yuan et al., 2007; Setchell and Clerici, 2010). In the last decade, a number of bacterial strains capable of producing equol have been identified from human and animal sources (Wang et al., 2005; Uchiyama et al., 2007; Maruo et al., 2008; Yokoyama and Suzuki, 2008; Yu et al., 2008; Tsuji et al., 2010). Nearly all those isolated so far fall into the family *Coriobacteriaceae* (Clavel et al., 2014), which includes a series of newly described, nutritionally-fastidious species that are difficult to isolate from other intestinal microbes. Some qPCR methods for detecting and quantifying *Coriobacteriaceae* species in faecal samples have already been developed (Harmsen et al., 2000; Thorasin et al., 2015; Cho et al., 2016). However, as the amplification primers are based on 16S rRNA sequences, their coverage is currently uncertain in the highly diverse human gut ecosystem.

The aim of the present work was to develop a qPCR method capable of identifying and quantifying equol-producing bacteria by targeting functional genes involved in the synthesis of this compound. This methodology would provide a new tool for evaluating strategies aimed to increase the endogenous formation of equol. The specificity and sensitivity of the method was tested using pure cultures of equol-producing and non-equol-producing strains of intestinal bacterial species. The method was then tested using human faecal samples, and the slurry cultures derived from them, provided by equol-producing and non-equol-producing women. Isoflavones and their metabolites were quantified by ultra-high performance liquid chromatography (UHPLC).

## Materials and methods

### Human intervention study

This study was approved by the Bioethics Committee of CSIC (Consejo Superior de Investigaciones Científicas) and by the Regional Ethics Committee for Clinical Research (Servicio de Salud del Principado de Asturias, Spain). The selection of donors and later sampling was performed following standardized protocols recommended by the above committees. All subjects gave written informed consent in accordance with the declaration of Helsinki. The faecal samples analysed in this work, or utilized as inoculants for the faecal cultures, had been collected in a previous intervention study of menopausal women under treatment with a soy isoflavone concentrate (Guadamuro et al., 2015). In short, participants (*n* = 18; age range 48–61 years, mean 52.6 years; body weight range 52–73 kilo; average 65.3) consumed for 6 months one tablet a day containing 80 mg of isoflavones containing genistin/daidzin in the range of 55–72% (Fisiogen; Zambon). Faeces were taken a three time points: before the start of the intervention (time 0), and at 1, 3, and 6 months of treatment. Freshly voided stools were collected in sterile plastic containers and transported to the laboratory, where they were kept frozen at −80°C until analysis.

### Bacteria and culture conditions

Table 1 shows the bacteria used in the present work. Strains of most species were obtained from the Leibniz Institut-Deutsche Sammlung von Mikroorganismen und Zellkulturen (DSMZ) collection. Intestinal species were cultured in either Gifu Anaerobic Medium (GAM) broth (Nissui Pharmaceuticals, Tokyo, Japan) supplemented with 0.5% arginine (Merck, Darmstad, Gemany) (GAM-Arg), or Reinforced Clostridium Medium (RCM) (Merck), at 37°C in a Mac500 anaerobic chamber (Down Whitley Scientific, West Yorkshire, UK) under anoxic atmospheric conditions (10% H_2_, 10% CO_2_, and 80% N_2_). *Escherichia coli*, however, was grown in Luria-Bertani (LB) broth in aerobiosis at 37°C with shaking.

**Table 1:** Bacterial strains and oligonucleotide primers utilized in this work.

**Strain/item**	**Phenotype/sequence**	**Origin/target (position)**	**Source/reference**
*Adlercreutzia equolifaciens* DSM 19450^T^	Equol-producer	Human faeces	DSMZ
*Asaccharobacter celatus* DSM 18785^T^	Equol-producer	Rat caecum	DSMZ
*Enterorhabdus mucosicola* DSM 19490^T^	Equol-producer	Ileal mucosa	DSMZ
*Slackia equolifaciens* DSM 24851^T^	Equol-producer	Human faeces	DSMZ
*Slackia isoflavoniconvertens* DSM 22006^T^	Equol-producer	Human faeces	DSMZ
*Bacteroides fragilis* DSM 2151^T^	Equol non-producer	Appendix abscess	DSMZ
*Bacteroides thetaiotaomicron* DSM 2079^T^	Equol non-producer	Human faeces	DSMZ
*Bifidobacterium longum* H66	Equol non-producer	Human faeces	Laboratory collection
*Blautia coccoides* DSM 935^T^	Equol non-producer	Mouse faeces	DSMZ
*Blautia producta* DSM 2950^T^	Equol non-producer	Human septicemia	DSMZ
*Blautia obeum* DSM 25238^T^	Equol non-producer	Human faeces	DSMZ
*Collinsella intestinalis* DSM 13280^T^	Equol non-producer	Human faeces	DSMZ
*Escherichia coli* A-15	Equol non-producer	Dairy biofilm	Laboratory collection
*Faecalibacterium prausnitzii* DSM 17677	Equol non-producer	Human faeces	DSMZ
*Lactobacillus rhamnosus* E41	Equol non-producer	Human faeces	Laboratory collection
*Prevotella copri* DSM 18205^T^	Equol non-producer	Human faeces	DSMZ
Oligonucleotide primers	(5′–3′)		
tdr.qPCR-F	RTYAACGGCRAYATGCAGGT	*tdr* (1279–1298)^a^	This work
tdr.qPCR-R	GGMAYYTCCATGTTGTAGGA	*tdr* (1372–1391)^a^	This work
ddr.qPCR-F	CTCGAYCTSGTSTACAACGT	*ddr* (421–440)^a^	This work
ddr.qPCR-R	GARTTGCAGCGRATKCCGAA	*ddr* (607–626)^a^	This work
dzr.qPCR-F	GAAGCTTGATATGGACGACT	*dzr* (669–688)^a^	This work
dzr.qPCR-R	GGAATATGCACCTGTTCCT	*dzr* (854–872)^a^	This work
TBA-F	CGGCAACGAGCGCAACCC	16S rRNA gene	Denman and McSweeney, 2006
TBA-R	CCATTGTAGCACGTGTGTAGCC	16S rRNA gene	Denman and McSweeney, 2006

*(^T^)Type strain*.

*DSMZ, Leibniz Institut-Deutsche Sammlung von Mikroorganismen und Zellkulturen, Braunschweig, Germany*.

a *According to the numbering of genes in S. isoflavoniconvertens DSM 22006^T^ (GenBank accession number JQ358709)*.

### Faecal cultures

Ten-fold faecal dilutions were prepared by homogenizing 1 g of faeces in 9 ml of pre-reduced phosphate buffer saline (PBS) in an anaerobic atmosphere (as above). A 10% (v/v) aliquot of the resulting faecal slurry was used to inoculate GAM-Arg to which the isoflavones daidzein or genistein (Toronto Research Chemical, Toronto, Canada) were added at a concentration of 100 μM. Faecal cultures were then incubated in open tubes under anaerobic conditions at 37°C for 24 h. They were then centrifuged at 13,500 rpm for 5 min, and the supernatants and pellets independently collected for isoflavone metabolite analysis and DNA extraction, respectively.

### Detection and quantification of isoflavone metabolites

Daidzein and genistein, and their metabolites dihydrodaidzein, dihydrogenistein, and equol, were measured in faecal cultures by a UHPLC procedure based on the method for determining isoflavones in urine (Redruello et al., 2015). After filtering through a 0.2 μm PTFE membrane (VWR, Radnor, PA, USA), culture supernatants were used directly (i.e., without purification or any extraction step) in UHPLC analysis. Cultures were analysed in duplicate. Quantification was performed against calibration curves prepared using commercially available standards.

### DNA extraction from bacteria, faecal samples, and faecal cultures

Total DNA from both control bacteria and microorganisms from faeces and faecal cultures was extracted following the procedure of Zoetendal et al. (2006) using the QIAamp DNA Stool Minikit (Qiagen, Hilden, Germany), with minor modifications as reported by Guadamuro et al. (2015). DNA was finally eluted with 150 μL sterile molecular biology grade water (Sigma-Aldrich, St. Louis, CA., USA) and stored at −20°C until use in real-time (qPCR) amplifications.

### Design of primers targeting genes involved in equol production

Genome sequences of equol-producing bacteria deposited in the NCBI database (http://www.ncbi.nlm.nih.gov/genome/) were downloaded. Sequences of the genes encoding reductases identified in equol-associated gene clusters [tetrahydrodaidzein reductase (*tdr*), dihydrodaidzein reductase (*ddr*) and daidzein reductase (*dzr*)] were then aligned using Clustal Omega software (http://www.ebi.ac.uk/Tools/msa/clustalo/) (Supplementary Figure 1). Degenerated oligonucleotide primers were manually designed based on the conserved regions of the genes (Table 1). The efficacy and specificity of the primers were evaluated using DNA from equol-producing and non-producing intestinal bacteria as a template (Table 1).

### Real-time qPCR

Real-time qPCR was performed using a 7,500 Fast Real-time PCR System running software version 2.0.4 (Applied Biosystems, Foster City, CA., USA). Amplification and detection were performed in 96-well optical plates (Applied Biosystems) with SYBR-Green (Applied Biosystems). All amplifications were performed in triplicate in a final volume of 20 μL containing 10 μL of a 2xSYBR Green PCR Master Mix including ROX as a passive reference (Applied Biosystems), 900 nM of each primer, and 2 μL of template DNA (5–10 ng). For amplification, the standard protocol of the 7,500 thermocycler (Applied Biosystems) was followed, i.e., an initial cycle at 95°C for 10 min, followed by 40 cycles at 95°C for 15 s, and 1 min at 60°C. To check for specificity, melting curve (Tm) analysis was performed, increasing the temperature from 60 to 95°C at a rate of 0.2°C per second with the continuous monitoring of fluorescence. Equol-producing bacteria in faecal samples were enumerated using standard curves for genes coding for reductases in equol-producing strains (Table 2). These curves were prepared using 10-fold serial dilutions of DNA extracted from cultures of equol-producing strains of known size (determined in GAM-Arg agar plates at 37°C after 72 h under anaerobic conditions). The absence of PCR inhibitors in negative samples was ruled out by amplifying prokaryotic 16S rRNA gene sequences using universal primers (Table 1). The efficiency of the equol-related primers was calculated from the slope of the standard curve for each primer set using the formula *E* = 10^−1/slope^. Positive amplification was deemed to have occurred when a Ct value of ≤30 was recorded; this corresponds to a total bacteria detection limit of <10^2^ cfu/ml (as determined by amplification with the 16S rRNA-encoding gene primers mentioned above; Table 1).

**Table 2:** Temperature of melting, efficiency and regression equation obtained for the amplification of equol-associated reductase genes with the primers designed in this study and using as a template purified DNA from equol-producing bacteria.

**Equol-producing organism/target gene**	**Melting temperature**	**Efficiency (R^2^)**	**Regression equation**
**TETRAHYDRODAIDZEIN REDUCTASE (*tdr*) Gene**			
*Adlercreutzia equolifaciens* DSM 19450^T^	83.85 ± 0.09	0.992	y = −0.2907x + 11.266
*Asaccharobacter celatus* DSM 18785^T^	83.61 ± 0.17	0.992	y = −0.3007x + 12.190
*Enterorhabdus mucosicola* DSM 19490^T^	82.97 ± 0.39	0.996	y = −0.2917x + 11.172
*Slackia equolifaciens* DSM 24851^T^	84.05 ± 0.16	0.993	y = −0.3170x + 11.200
*Slackia isoflavoniconvertens* DSM 22006^T^	83.39 ± 0.19	0.994	y = −0.3040x + 11.110
**DIHYDRODAIDZEIN REDUCTASE (*ddr*) Gene**			
*A. equolifaciens* DSM 19450^T^	88.98 ± 0.23	0.974	y = −0.2662x + 11.296
*A. celatus* DSM 18785^T^	88.32 ± 0.09	0.994	y = −0.2729x + 11.780
*E. mucosicola* DSM 19490^T^	88.47 ± 0.23	0.988	y = −0.2727x + 11.252
*S. equolifaciens* DSM 24851^T^	88.29 ± 0.14	0.992	y = −0.2716x + 10.905
*S. isoflavoniconvertens* DSM 22006^T^	88.34 ± 0.20	0.998	y = −0.2946x + 10.821
**DAIDZEIN REDUCTASE (*dzr*) Gene**			
*S. equolifaciens* DSM 24851	-	-	-
*S. isoflavoniconvertens* DSM 22006^T^	85.91 ± 0.36	0.999	y = −0.3065x + 10.998

*y, values of log (cfu/ml); x, values of Ct. −,primers gave no amplification when DNA from S. equolifaciens was used as a template*.

### Statistical analysis

qPCR data were analysed using free R software (http://www.r-project.org). The Shapiro-Wilk test was used to check for the normal distribution of the data. The non-parametric Spearman rank correlation test was used to examine the relationship between the copy numbers of *tdr* and *ddr*, and between gene copy number and equol production. The Wilcoxon signed-rank test was used to determine whether the copy number of *tdr* or *ddr* genes differed over isoflavone treatment (0, 1, 3, and 6 months). The same test was used to correlate gene copy numbers and equol production. Significance was set at *P* < 0.05.

## Results

Alignment of the database-available reductase-encoding genes showed the *tdr* and *ddr* sequences from the different species and strains shared sufficient nucleotide identity (71 and 78%, respectively; Supplementary Figure 2) to allow the design of “universal equol-related” primers (Table 1). However, the *dzr* genes were so divergent (36% nucleotide identity only) that no primers could be designed that could detect all sequences. In an attempt to obtain at least some partial information on this gene, a pair of primers (Table 1) based on the sequence of the *dzr* genes from the two *Slackia* strains available -*S. isoflavoniconvertens* DSM 22006^T^ (Schröder et al., 2013) and *Slackia* spp. NATTS (Tsuji et al., 2012)—were synthesized.

**Figure 1:**
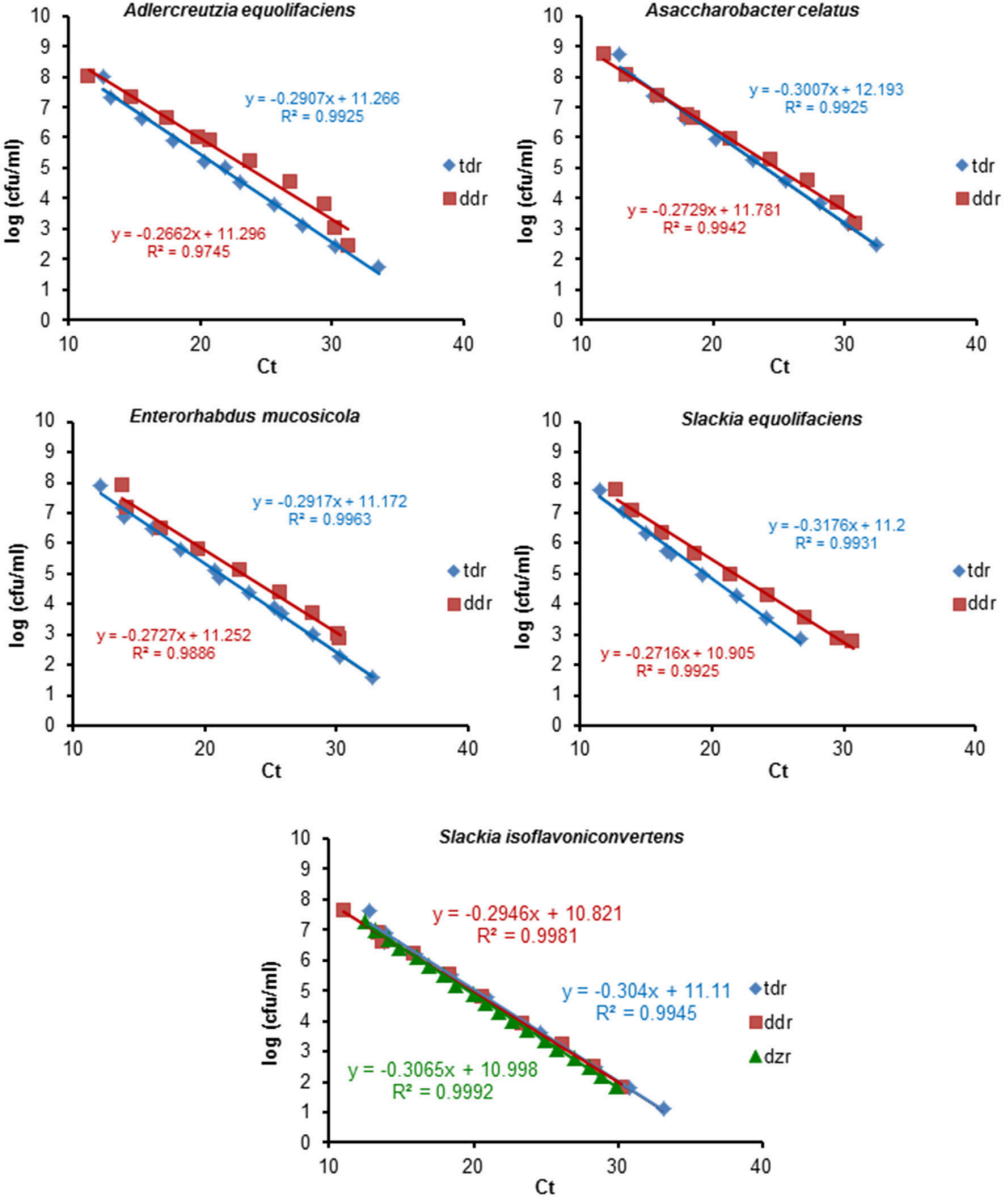
Standard curves of qPCR for the tetrahydrodaidzein reductase (*tdr*) and the dihydrodaidzein reductase (*ddr*) target genes using serial dilutions of DNA from known amounts of cells of equol-producing microorganisms. Linear regression was obtained plotting the cycle threshold (Ct) values vs. the log_10_ of the counting results (in cfu/ml). The equation and R^2^ value of the regression lines are indicated in each panel.

The specificity of the primers was experimentally tested against purified DNA from pure cultures of strains belonging to 16 representative bacterial species from the human gut (Table 1). Positive and negative qPCR assays corroborated *in silico* predictions. Amplification was only obtained when DNA from equol producing strains was used as a template (Table 1). Table 2 summarises the key parameters of the amplification reactions using the three pairs of primers developed. Figure 1 shows the standard curves for the qPCR detection of *tdr, ddr* and *dzr*, prepared using serial dilutions of DNA containing known numbers of equol-producing microorganisms. Linear regressions were obtained by plotting the cycle threshold (Ct) values against the log_10_ enumeration values for the equol producing strains (in cfu/ml). The detection limit of the qPCR assay was determined using genomic DNA from these cultures, assuming a genome size of about 3.0 fg of DNA per cell (Rodríguez-Lázaro et al., 2004). Three independent experimental assessments of the detection limit of the qPCR reaction determined a sensitivity of 1–10 equol-producing bacteria or genome equivalents, giving a detection limit of about 10^2^ cfu/g of faeces. As shown in Table 2, the efficiency of the primer pair for amplifying the *dzr* gene was adequate when DNA from *S. isoflavoniconvertens* was used as a template, but not when the DNA came from *S. equolifaciens*. This pair of primers was, therefore, no longer used.

**Figure 2:**
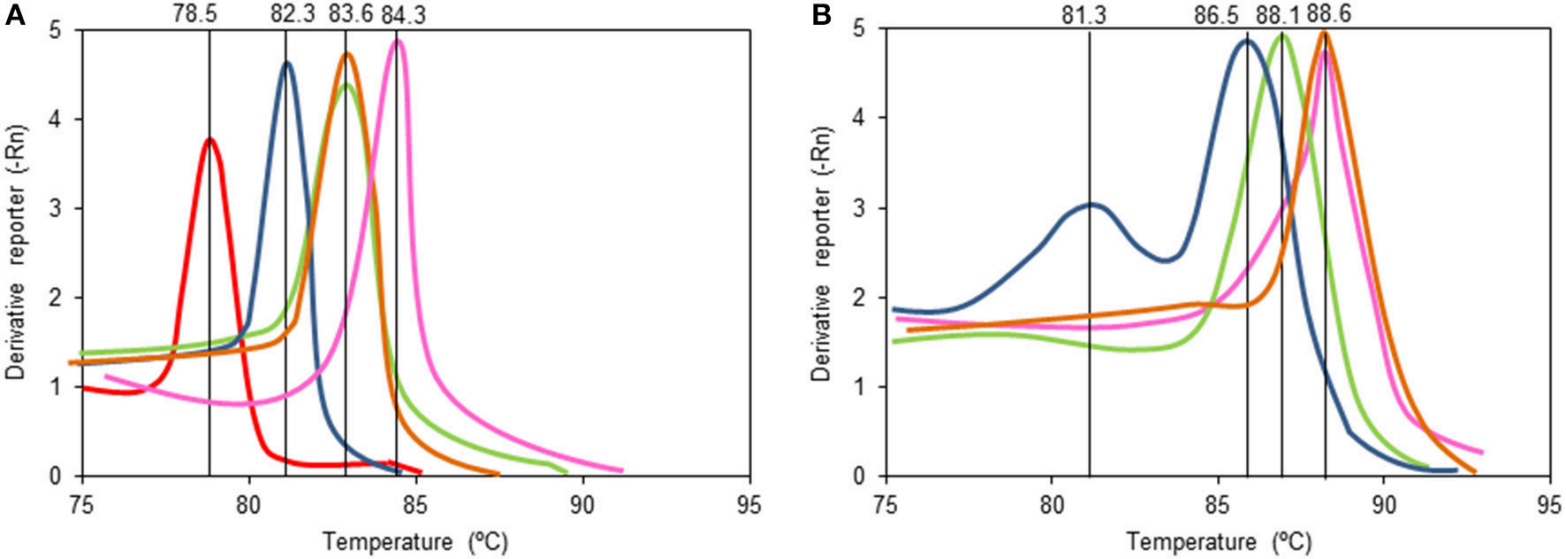
Melting curves of qPCR amplicons with the primers designed in this study targeting the tetrahydrodaidzein reductase (*tdr*) gene **(A)** and dihydrodaidzein reductase (*ddr*) **(B)** gene using as a template total microbial DNA from cultures containing daidzein inoculated at 1% with feces from equol producing women W3 (in green), W8 (in blue), and W18 (in red) and no-equol producing women W7 (in orange) and W15 (in pink). Note that the ddr gene gave no amplification in cultures derived from samples of woman W18 **(B)**.

The primers for *tdr* and *ddr* were then used to explore DNA purified from the faecal samples of the 18 isoflavone-treated menopausal women before starting isoflavone intake (time 0) and after 1, 3, and 6 months of treatment. Three of these women (W3, W8, and W18) had previously been shown to have an equol-producing phenotype based on their urine equol/creatinine ratios of >5.0 (Guadamuro et al., 2015). Table 3 shows the results of the amplification, based on the Ct value, and the calculated absolute abundance of equol-producing organisms. Negative amplification (absence of target genes) was accepted when the Ct was >30. As expected, *tdr* was detected in all samples provided by the equol-producing women. However, the *ddr* gene was identified in the faeces of only two (W3 and W8) of the three equol producers, while no amplification was obtained for this gene when used as template DNA for examining the faeces and faecal slurry cultures of subject W18. Changes in the Ct values of samples from each of the women were observed over the isoflavone treatment period, but with no particular trend apparent. Indeed, Wilcoxon analysis of the values at different time points revealed no significant differences (Supplementary Figure 3). It is noteworthy that when positive amplifications were detected for *tdr* and *ddr*, equivalent copy numbers were always observed (Spearman coefficient 0.918). As a pattern of the Tm curve in qPCR amplicons correlates with specific nucleotide sequences of amplicons, analysis of the Tm curves provides information on the number of sequences amplified in the reaction and their relationships. In this sense, analysis of the Tm curves (Figure 2) showed some amplicons (those of the *tdr* genes from samples provided by subjects W3 and W8, and that of *ddr* from subject W3) to have Tm patterns similar to those of equol-producing control bacteria (Table 2). In contrast, the Tm of other amplicons (*tdr* from W18 and *ddr* from W8) were rather different to those of the positive strains. Moreover, the *ddr* amplicon from W8 showed two separate peaks, indicating that two DNA fragments with different sequence are being amplified.

**Table 3:** Cycle threshold (Ct) values obtained in faecal samples for tetrahydrodaidzein reductase (*tdr*) and dihydrodaidzein reductase (*ddr*) genes and absolute abundance of equol producing bacteria in the real-time PCR assay developed in this study.

**Women**	**Sample (time)^a^**	**qPCR amplification of total microbial DNA from faeces**			
		**Ct (*tdr*)**	**Log_10_ (cfu/ml) ±SD**	**Ct (*ddr*)**	**Log_10_ (cfu/ml) ± SD**
**EQUOL PRODUCERS**					
W3	0	25.95 ± 0.01	3.57 ± 0.54	25.45 ± 0.05	4.17 ± 0.58
	1	23.75 ± 0.70	4.23 ± 0.53	25.00 ± 0.40	4.30 ± 0.58
	3	24.98 ± 0.18	3.86 ± 0.54	24.90 ± 0.04	4.32 ± 0.58
	6	26.24 ± 0.04	3.48 ± 0.54	25.91 ± 0.01	4.05 ± 0.58
W8	0	22.53 ± 0.14	4.60 ± 0.52	22.25 ± 0.03	5.06 ± 0.55
	1	22.86 ± 0.22	4.50 ± 0.52	22.53 ± 0.19	4.98 ± 0.55
	3	22.53 ± 0.22	4.60 ± 0.52	21.67 ± 0.04	5.22 ± 0.55
	6	22.53 ± 0.44	4.60 ± 0.52	21.43 ± 0.18	5.28 ± 0.55
W18	0	28.17 ± 0.57	2.90 ± 0.56	-	<2
	1	24.80 ± 0.22	3.92 ± 0.54		
	3	26.02 ± 0.25	3.55 ± 0.54		
	6	26.11 ± 0.22	3.52 ± 0.54		
**EQUOL NON-PRODUCERS**					
W1	0	-	<2	-	<2
	1				
	3				
	6				
W2	0	-	<2	-	<2
	1				
	3				
	6				
W4	0	-	<2	-	<2
	1				
	3				
	6				
W5	0	-	<2	-	<2
	1				
	3				
	6				
W6	0	-	<2	-	<2
	1				
	3				
	6				
W7	0	22.49 ± 0.08	4.61 ± 0.52	23.42 ± 0.05	4.73 ± 0.56
	1	23.40 ± 0.02	4.34 ± 0.53	24.30 ± 0.13	4.49 ± 0.57
	3	23.57 ± 0.13	4.29 ± 0.53	24.60 ± 0.01	4.41 ± 0.57
	6	28.01 ± 0.51	2.95 ± 0.56	27.56 ± 0.08	3.59 ± 0.57
W9	0	-	<2	-	<2
	1				
	3				
	6				
W10	0	-	<2	-	<2
	1				
	3				
	6				
W11	0	-	<2	-	<2
	1				
	3				
	6				
W12	0	-	<2	-	<2
	1				
	3				
	6				
W13	0	-	<2	-	<2
	1				
	3				
	6				
W14	0	-	<2	-	<2
	1				
	3				
	6				
W15	0	24.16 ± 0.12	4.11 ± 0.53	25.77 ± 0.15	4.09 ± 0.52
	1	23.22 ± 0.08	4.39 ± 0.53	26.28 ± 0.25	3.94 ± 0.52
	3	23.91 ± 0.16	4.18 ± 0.53	27.18 ± 0.14	3.70 ± 0.53
	6	25.78 ± 0.12	3.62 ± 0.54	26.64 ± 0.02	3.84 ± 0.53
W16	0	-	<2	-	<2
	1				
	3				
	6				
W17	0	-	<2	-	<2
	1				
	3				
	6				

*−, qPCR negative (Ct > 30.00)*.

a *Samples were taken before the start of 0 and at 1, 3, and 6 months during isoflavone treatment*.

It was also surprising to find amplicons of both *tdr* and *ddr* when examining the samples of two non-equol-producing women (W7 and W15) (Table 3). To confirm that the phenotypic results of equol production (whether positive and negative) had been maintained, and to gain further insights into the metabolism of soy isoflavones, faecal slurries from selected faecal samples provided by equol-producing and non-equol-producing women (including those in which *tdr* and *ddr* were detected) were inoculated into GAM-Arg medium with no isoflavones (control) or with either daidzein (DZEN) or genistein (GTEN), and incubated for 24 h at 37°C under anaerobic conditions. DNA isolated from the faecal cultures was then subjected to qPCR analysis for the detection and quantification of *tdr* and *ddr* under the same conditions as above. In addition, isoflavones and their metabolites, including daidzein, genistein, dihydrodaidzein, dihydrogenistein, and equol, were measured in the faecal cultures by UHPLC. Table 4 shows the results obtained. No isoflavones or their metabolites were ever detected in the control cultures without added isoflavones. When daidzein and genistein were added to the faecal slurry cultures they were recovered from the uninoculated samples in varying amounts (67–81% of the added amounts). However, the added daidzein completely disappeared (converted into equol) when the medium containing this isoflavone was inoculated with *S. isoflavoniconvertens* DSM 22006. Equol in the faecal cultures was only present in those provided by the equol-producing women. However, the transformation of daidzein into equol was never complete, and variable amounts of daidzein (and usually dihydrodaidzein) were recovered from these cultures. Variable amounts of genistein and its derived metabolite dihydrogenistein were also recovered from cultures when this isoflavone was added. The exception was the faecal culture from W8, in which no genistein (detection limit 15.17 nM [Redruello et al., 2015]) and only a small quantity of dihydrogenistein (0.41 μM), was scored.

**Table 4:** Cycle threshold (Ct) values obtained by qPCR for tetrahydrodaidzein reductase (*tdr*) and dihydrodaidzein reductase (*ddr*) genes and isoflavone metabolites in the faecal slurry cultures.

**Faecal sample**	**GAM-Arg with^a^**	**qPCR amplification from faecal cultures**		**Isoflavone metabolites in faecal cultures (in μM)**				
		**Ct (*tdr*)**	**Ct (*ddr*)**	**Daidzein**	**Dihydrodaidzein**	**Genistein**	**Dihydrogenistein**	**Equol**
**EQUOL PRODUCERS**								
W3.3	Control	25.28 ± 0.07	26.07 ± 0.14	-	-	-	-	-
	DZEN	23.57 ± 0.17	24.60 ± 0.08	43.03	28.28	-	-	10.99
	GTEN	26.30 ± 0.02	26.84 ± 0.04	-	-	24.42	22.36	-
W8.1	Control	25.34 ± 0.38	23.47 ± 0.17	-	-	-	-	-
	DZEN	22.32 ± 0.31	21.10 ± 0.07	49.45	0.87	-	-	10.69
	GTEN	21.65 ± 0.43	20.78 ± 0.17	-	-	-	0.41	0.15
W18.1	Control	28.67 ± 0.55	-	-	-	-	-	-
	DZEN	29.56 ± 0.43	-	54.36	2.27	-	-	11.01
	GTEN	28.95 ± 0.20	-	-	-	57.46	2.15	-
**EQUOL NON-PRODUCERS**								
W1.3	Control^a^	-	-	-	-	-	-	-
	DZEN	-	-	69.54	-	-	-	-
	GTEN	-	-	-	-	16.30	0.94	-
W5.3	Control	-	-	-	-	-	-	-
	DZEN	-	-	42.22	37.80	-	-	-
	GTEN	-	-	-	-	15.40	23.95	-
W7.3	Control	22.52 ± 0.15	23.24 ± 0.16	-	-	-	-	-
	DZEN	25.61 ± 0.07	26.35 ± 0.04	80.32	-	-	-	-
	GTEN	24.86 ± 0.01	25.70 ± 0.04	-	-	2.48	83.17	-
W15.3	Control	25.81 ± 0.04	26.90 ± 0.02	-	-	-	-	-
	DZEN	25.41 ± 0.09	26.26 ± 0.10	72.03	1.14	-	-	-
	GTEN	25.62 ± 0.11	26.54 ± 0.04	-	-	5.81	50.80	-
W17.1	Control	-	-	-	-	-	-	-
	DZEN	-	-	72.37	9.19	-	-	-
	GTEN			-	-	47.77	5.98	-
**CULTURE CONTROLS**								
*S. isoflav*^b^	Control	nd	nd	-	-	-	-	-
	DZEN	nd	nd	-	-	-	-	100.00
	GTEN	nd	nd	-	-	45.76	6.69	0.79
GAM-Arg^c^	Control	nd	nd	-	-	-	-	-
	DZEN	nd	nd	81.18	-	-	-	-
	GTEN	nd	nd	-	-	67.00	-	-

*-, qPCR negative (Ct > 30.00) or isoflavone metabolite under the limit of detection; nd, not done*.

a *The medium used for the faecal cultures (GAM-Arg) contained either daidzein (DZEN), genistein (GTEN), or no isoflavones (Control)*.

b *S. Isoflav, culture of Slackia isoflavoniconvertens DSM 22006 in GAM-Arg*.

c *GAM-Arg, uninoculated culture medium incubated under the same conditions*.

The qPCR results obtained for the faecal cultures matched those obtained with DNA isolated from faeces. The data agreed well both qualitatively and quantitatively, with the Ct values of the corresponding faeces and faecal culture samples following the same trend. The *tdr* gene was shown to be present in cultures inoculated with faecal material from all three equol-producing women (W3, W8, and W18), while *ddr* was only detected in the cultures from subjects W3 and W8. Once again, both *tdr* and *ddr* were identified in cultures from the non-equol-producing subjects W7 and W15.

## Discussion

Conventional means of identifying and quantifying microorganisms in complex ecosystems, such as those in the gastrointestinal tract, are laborious, time consuming, and only recover the cultivable part of their populations (Qin et al., 2010; Browne et al., 2016). Molecular, culture-independent microbial techniques, such as qPCR, are therefore essential if gut-dwelling microorganisms are to be reliably identified and quantified. This is particularly important when tracking microorganisms involved in intestinal functionality, such as those that produce equol (Clavel et al., 2014). Strain-specific oligonucleotide primers based on 16S rRNA gene sequences for the equol-producing *Slackia* spp. NATTS have already been reported (Tsuji et al., 2010; Sugiyama et al., 2014). 16S rRNA gene-based primers targeting *Coriobacteriaceae* species have also been developed (Harmsen et al., 2000; Thorasin et al., 2015; Cho et al., 2016). However, as the microbial typing of the human gut microbiota is not yet complete (Harmsen and de Goffau, 2016), the coverage of the current coriobacterial primers is uncertain. Further, whether equol-production is a phylogeny-related trait (species-specific) or an acquired property (strain-specific) has yet to be determined (Clavel and Mapesa, 2013). Thus, primers targeting functional, single-copy genes, such as those involved in equol biosynthesis, are preferable.

The present work reports a qPCR assay involving oligonucleotide primers based on conserved sequences of reductase-encoding genes implicated in the synthesis of equol, plus the use of SYBR Green as a dye, for the detection and quantification of equol-producing bacteria. The main advantage of using SYBR Green instead of molecular probes is its lower cost and a reduced need for optimisation (Inglis and Kalischuk, 2004). The specific binding of SYBR Green to any double stranded nucleic acid allows the detection of non-specific and/or multiple amplifications by Tm curve analysis. The proposed qPCR assay clearly distinguished target species from all non-target species analysed belonging to the same ecosystem, thus demonstrating its specificity. It also showed excellent quantification characteristics in terms of both linear dynamic range and relative accuracy. In addition, it performed equally well with purified DNA from equol-producing bacteria and total microbial DNA from faeces and faecal slurry cultures. Unfortunately, since only limited information on the microbial types involved in the synthesis of equol is available, the identification and quantification of equol producers by qPCR technique developed in this work might be compromised. This could result in the underestimation of the amount of DNA present (and thus the number of equol-producing bacteria), or even in amplification failure. Sequences of genes from newly discovered equol-producing bacteria could be incorporated into the design of “universal”, “group-specific,” or “species-specific” primer pairs to improve the sensitivity, coverage and accuracy of the proposed assay. It is interesting to note that, when positive amplification of *tdr* and *ddr* was observed, equivalent copy numbers of these two genes were recorded, suggesting they are located in the same genetic element (such as in the chromosome in an operon-like structure for *S. isoflavoniconvertens*; Schröder et al., 2013; Tho et al., 2013). The fact that the copy number of the genes did not increase significantly over the course of subjects' treatment suggests that equol-producing bacteria are not being positively selected by the short-term isoflavone intake assessed in this study. The slight increase in *Coriobacteriaceae* members seen during isoflavone consumption (Nakatsu et al., 2014; Guadamuro et al., 2015) might be a consequence of the inhibition of dominant intestinal bacterial populations by these compounds or their metabolites.

Analysis of the Tm amplification curves suggests the presence in the faecal samples of some women of gene sequences identical or very similar to those of the equol-producing bacterial species used as a control. However, in other subjects, Tm values different to those of the controls were also observed, indicating the presence of genes in their faeces with nucleotide differences, which suggests the involvement of unrelated taxa in equol formation in the faeces of these individuals. The presence of two equol-producing species might be responsible for the two peaks observed in the Tm of samples from subject W8 -although, non-specific amplification would also give rise to the same results. Sequencing and analysis of amplicons obtained through conventional PCR using primers targeting other conserved regions of both *ddr* and *tdr* genes further support the presence of not yet reported genes in some faecal samples (data not shown). An unrelated *ddr* gene might also be carried by subject W18, for whose samples no amplification of this gene was ever recorded. Altogether, these observations suggest that equol-associated genes (and thus equol-producing bacteria) unrelated to those reported in the literature (Jin et al., 2010; Shimada et al., 2010, 2012; Tsuji et al., 2012; Schröder et al., 2013; Tho et al., 2013) might be present in the gut of the subjects of this study.

Among the inconsistencies regarding the detection of genes and equol production, it is noteworthy the positive amplification of both *tdr* and *ddr* in DNA from faeces and slurry cultures of two non-equol-producing women. The presence of non-functional, yet amplifiable genes, the conversion of daidzein into downstream metabolites other than equol, and the presence of homologous genes encoding enzymes without activity over daidzein, could account for this apparent contradiction. However, the addition of equol to faecal cultures from subjects W7 and W15, excluded the possibility of any further metabolism of this compound (data not shown). The presence of related genes might be supported by the recovery of a large proportion of the genistein in the faecal cultures of these two women as dihydrogenistein (Table 4). From a chemical point of view, daidzein and genistein are highly similar molecules (del Rio et al., 2013), which suggests that enzymes acting on these compounds might show structural and functional similarities. Indeed some enzymes, as has been demonstrated for the reductases of *S. isoflavoniconvertens*, act on both daidzein and genistein aglycones (Matthies et al., 2012). Therefore, the presence of genes coding for enzymes acting in genistein only but with enough nucleotide identity to be amplified with the primers of this study without equol production is plausible. If this were the case, it would result in DNA (and thus the number of equol-producing bacteria) overestimation.

In conclusion, this work reports a highly specific, sensitive and reliable qPCR assay for the detection and quantification of equol-producing bacteria in microbiologically complex samples, including human-derived faecal samples and faecal cultures. Combining the qPCR technique described here with the detection and quantification of equol in biological fluids (urine) and culture supernatants by chromatographic methods would help tracking equol-producing populations in the gut. Monitoring equol-producing microorganisms in the human gut could provide a means of evaluating strategies aimed at increasing the endogenous formation of this compound. The biological significance of the presence/absence of *tdr* and *ddr* genes in isoflavone metabolism and equol production is currently under study.

## Author contributions

BM and AF conceived the study. LV and LG were involved in the experimental determinations. FG engaged participants and provided samples. BM provided materials and resources. AF drafted the manuscript. BM made a critical revision of the manuscript. All authors reviewed and approved the final version.

### Conflict of interest statement

The authors declare that the research was conducted in the absence of any commercial or financial relationships that could be construed as a potential conflict of interest.
