# Supplementary figures and images for: Development and Use of a Real-Time Quantitative PCR Method for Detecting and Quantifying Equol-Producing Bacteria in Human Faecal Samples and Slurry Cultures
Source: Front Microbiol. 2017 Jun 30;8:1155. doi: 10.3389/fmicb.2017.01155 (PMC5491606; doi:10.3389/fmicb.2017.01155)

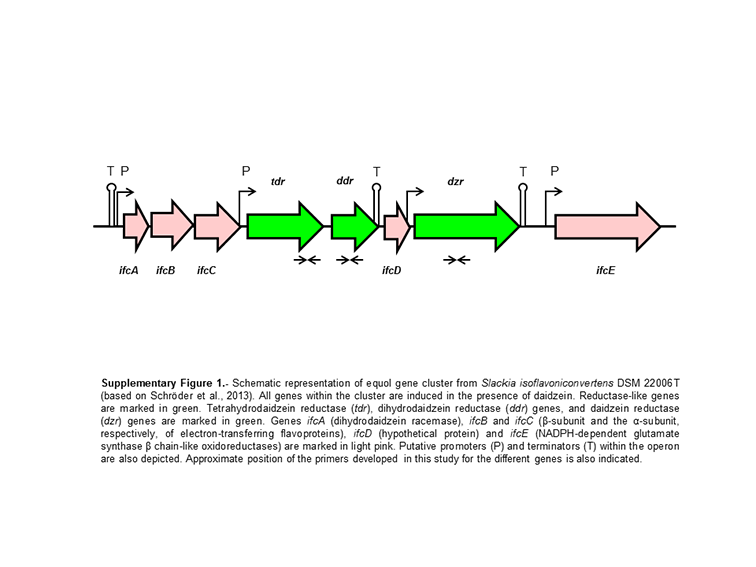

Supplement: Supplementary file 1 [file Image1.tif]

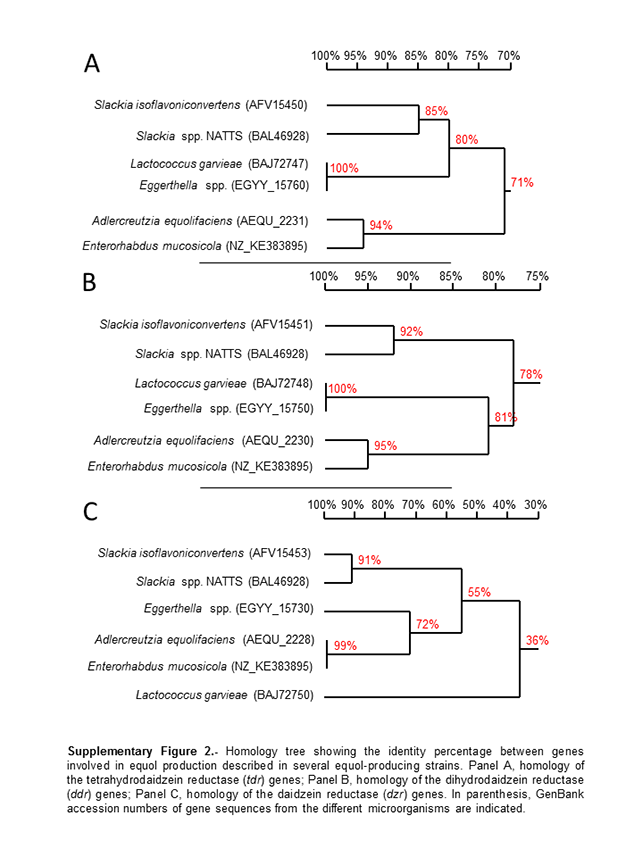

Supplement: Supplementary file 2 [file Image2.TIF]

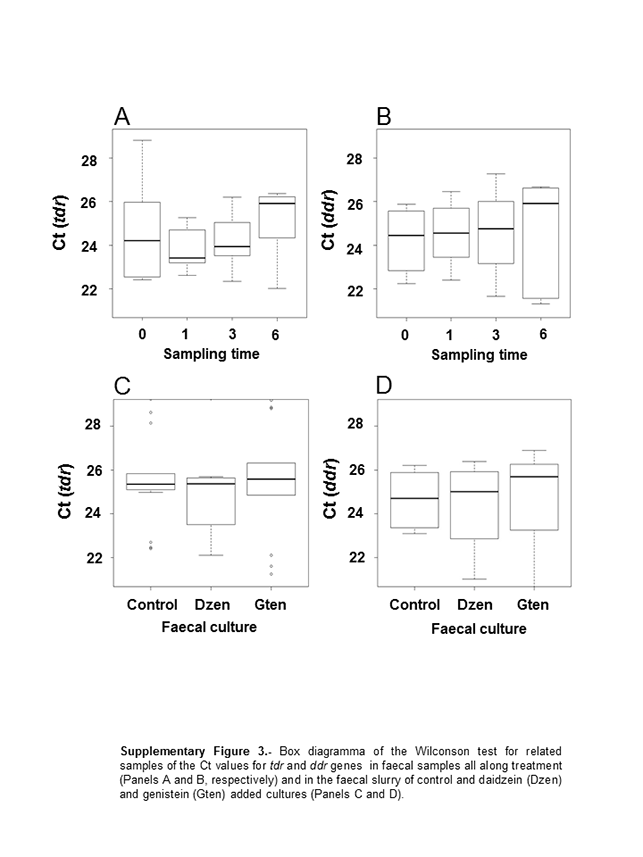

Supplement: Supplementary file 3 [file Image3.tif]
